# Parliament and the Professions

**Published:** 1920-03-20

**Authors:** 


### Parliament and the Professions.

### Medical Remuneration under National Health Act.

Dr. Addison informed Sir Philip Magnum that after arbitration it had been found that 11s. per insured person to be made available to provide payment for medical men under the National Health Insurance Service Scheme is a sum that should properly be paid to them. The Minister could not give details as to what the gross cost of this increase would be.

### Neurasthenic Ex-soldiers.

The Minister of Pensions stated that there are twelve special institutions, with accommodation for nearly 1,500 patients, available for the treatment of neurasthenic cases. A considerable number of beds are also reserved for such cases at other Ministry hospitals. These institutions are all under the direct control of the Ministry, and are unconnected with lunacy administration.

### Venereal Disease.

The Minister of Health informed Mr. J. Davison that records are kept regarding the treatment centres for venereal diseases established partly or wholly at the public expense. The number of cases dealt with for the first time at these centres between January 1, 1917, when the first treatment centres were opened, and December 31, 1919, is approximately 175,000. The estimated expenditure during the current financial year is £314,000, 75 per cent. of which is borne by national funds and 25 per cent. by local funds.

